# Hydraulic Characterization of Ceramic Foam Filters Used in Aluminum Filtration

**DOI:** 10.3390/ma16072805

**Published:** 2023-03-31

**Authors:** Massoud Hassanabadi, Thomas Berto, Shahid Akhtar, Ragnhild E. Aune

**Affiliations:** 1 Department of Materials Science and Engineering, Norwegian University of Science and Technology (NTNU), 7491 Trondheim, Norway; thomas.brt@hotmail.it (T.B.); ragnhild.aune@ntnu.no (R.E.A.); 2 Hydro Aluminum, Romsdalsvegen 1, 6600 Sunndalsøra, Norway; shahid.akhtar@hydro.com

**Keywords:** aluminum refining, aluminum recycling, CFF, permeability, pressure drop, Darcy law

## Abstract

Ceramic Foam Filters (CFF) are frequently used during the filtration of aluminum (Al) melts to produce high-quality products. In the present study, the physical and hydraulic characteristics of alumina (Al_2_O_3_)-based CFF from three different suppliers (A, B and C) have been thoroughly investigated. The filters’ porosity and pore diameter, i.e., *Window* and *Cell Feret* diameters, were measured and the permeability of the different filters calculated based on pressure drop experiments. The comparison of the classification systems of CFF, i.e., Grade and PPI (Pore Per Inch) numbers, using statistical analysis of permeability and *Window Feret* diameter showed significant variations between the morphological and hydraulic properties of some CFFs of identical Grade and PPI numbers. Moreover, the *Fanning* friction factor was plotted as a function of interstitial Reynolds numbers (*Re_i_*), and laminar, transient, and turbulent flow regimes were identified. The relationship between the *Fanning* friction factor and the interstitial Reynolds numbers of all the filter samples investigated was processed using regression analysis, and a model equation developed to calculate the pressure drop over the CFF using the *Window Feret* diameter. The correlation between the experimental pressure drop values and the derived model equation indicates that empirical expressions for calculating the pressure drop over CFFs should be derived based on experimental measurements carried out at the velocity range of the application of the CFF, which is about 10 mm·s^−1^ for aluminum filtration.

## 1. Introduction

The filtration of aluminum (Al) using alumina (Al_2_O_3_) Ceramic Foam Filters (CFF) has been an approach extensively used in Al cast houses since their invention in 1974 [1]. Through the filtration process, particles/inclusions, i.e., metallic, and non-metallic impurities, are collected on or in the filter [2]. CFFs are, however, wetted unfavorably by molten Al, so a metallic static head is required to prime the filters, i.e., the force needed to push the air inside the tortuous structure of the filters out, and subsequently infiltrate the porous material [1,3]. The filter capacity depends on the type and size of the particles collected [4]. The filtration modes, i.e., cake and/or depth filtration, as well as the flow velocity, play significant roles in the removal of the particles [5]. Depth filtration, i.e., removal of particles on the filter walls, is more desired than cake filtration, especially if the size of the particles is larger, as they may clog the filter [6]. Larger particles, as well as the number of oxide films present in the molten metal, can be reduced both in size and amount through a pre-refining process, i.e., fluxing, degassing, or filtration using Fiber glass cloth filters in the launder, making it possible to efficiently use cake filtration [7].

Interception and gravity are the main collision mechanisms through which the particles are trapped on the walls of the CFF [8]. Due to the higher interfacial tension of the filter material relative to the melt, the collection efficiency is not equal to the collision efficiency, and particles are partly collected and later re-entrained in the melt [9]. This phenomenon escalates with a more significant pressure drop and/or velocity [5], and the optimum conditions for molten metal filtration should, therefore, be considered through which the re-entrainment forces, i.e., viscous drag and inertial forces, are less than the restraining forces [2]. 

Through the semi-continuous casting process of Al, i.e., through Direct Chill (DC) casting, the filtration is performed at flow rates from 5 to 1200 kg·min^−1^ (≈10 mm·s^−1^) for filters of Grade 10–80 [1]. The different flow regimes induced in the CFF relative to the flow velocity, i.e., laminar, transient, and turbulent flow regimes, can affect the filtration efficiency as the transition from one flow regime to another, e.g., from laminar to the transient flow regime, persuade inertial forces on the trapped particles that may result in re-entrainment of the particles into the flow of molten metal [2]. Therefore, estimating the onset of the transient flow regime through the CFF of various Grades or Pores Per Inch (PPI) is essential to define the induced forces on the particles. Nevertheless, the studies that have been performed to characterize and determine the hydraulic properties of alumina CFF for aluminum filtration applications have been performed in the velocity ranges >> 15 mm·s^−1^ [10,11,12,13,14,15]. In addition, the CFF from various suppliers are named according to two different systems, i.e., Grade and PPI. The PPI number is traditionally used to characterize the CFF [1]. In a grading system, a CFF Grade 20 has a pore (*Cell*) size of approximately half that of a CFF Grade 10, and a CFF Grade 40 is half of a CFF Grade 20, etc. [16]. The general idea is that a CFF of, for instance, Grade 30 is equivalent to 30 PPI. However, due to different characterization methods, the morphology characteristics, hydraulic properties, and filtration efficiency would not be necessarily identical. In this regard, a thorough investigation of the different CFFs available for Al filtration is needed to define the flow regimes through the filters, i.e., for CFF of different Grades/PPI numbers. By establishing the characteristic length scales of the different filters, i.e., the *Cell* and *Window* sizes, as well as the permeability and the form drag coefficient, a uniform grading of the filters should be possible to obtain. 

The main objective of the present work is, therefore, to perform a fundamental characterization study of commercial CFF used in the Al filtration process. In view of this, the present study investigates the morphological characteristics and the pressure gradient of CFF of various Grades/PPI numbers from three different suppliers, as well as determines the dominating flow regimes within the different filters. An empirical equation was derived by correlation between experimentally obtained permeability values and the interstitial Reynolds numbers (*Re_i_*) to determine the pressure drop of different CFFs using the morphology characteristics, i.e., *Windows*’ mean *Feret* diameter, of the CFFs. 

## 2. Theory

**Figure 1:**
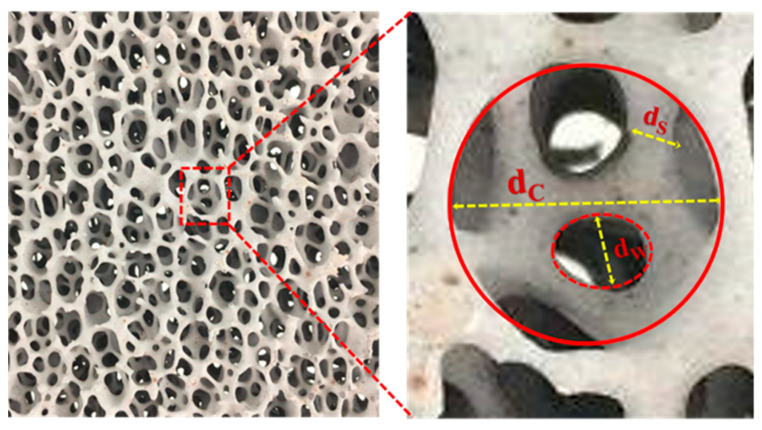
An uncut surface of a CFF of Grade 30, and the characteristic length scales, i.e., *Cell* (*d_c_*), *Window* (*d_w_*), and *Strut* (*d_s_*), which are indicated by the large solid circle, dotted circle, and double line arrow, respectively, in the enlarged section of image.

The present study uses the *Re* number, a dimensionless number expressing the inertia ratio to viscous forces, to identify the laminar, transition, and turbulent flow regimes in conduits [17]. By analogy, Reynolds number is also defined for flow through porous media [17]:

(1) $$Re_{i}=\frac{\rho du}{\mu }$$

where *d* is the length dimension of the porous media (m), *ρ* the fluid density (kg·m^−3^), *u* the superficial velocity (m·s^−1^), and *µ* the dynamic viscosity (Pa·s). The mean grain diameter is often taken as the length dimension (characteristic length scale) in unconsolidated porous media such as packed beds [18]. However, in CFFs with a network structure of polyhedral cells, it is customary to take the mean of the characteristic length scales, i.e., of the *Cell* (*d_C_*), *Window* (*d_w_*), and *Strut* (*d_s_*), as the length dimension [18,19], see Figure 1. Continuing the analogy with the flow in conduits, the flow in porous media could be expressed using a relationship between different friction factors and *Re* number [17]. The most common friction factor relations in cylindrical pipes are the *Darcy*–*Weisbach* and *Fanning* friction factors [17]:

(2) $$f=\frac{2d\Delta P}{L\rho u^{2}}\;\mathrm{Darcy}-\mathrm{Weisbach}$$

(3) $$f=\frac{d\Delta P}{2L\rho u^{2}}\;\mathrm{Fanning}$$

where *f* is the friction factor (dimensionless), *d* the diameter of the pipe (m), Δ*P·L*^−1^ the pressure loss per unit length (Pa·m^−1^), *ρ* the fluid density (kg·m^−3^), and *u* the mean flow velocity (m·s^−1^).

**Figure 2:**
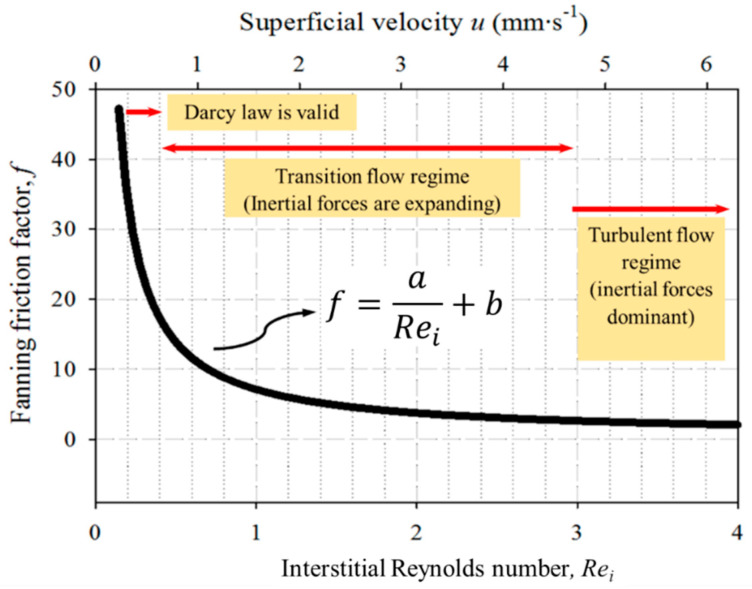
Schematic classification of the flow through a porous medium. Based on reference [17].

When considering flows through a porous medium, *d* is the same representative length as in Equation (1), and *u* the superficial velocity. The friction factor can be calculated using pressure drop experiments and plotting the result as a function of interstitial Reynolds number (*Re_i_*)) and the superficial velocity will generate a graph in which the existence of three different flow regions can be distinguished. Figure 2 illustrates a schematic of this graph with the three flow regions as follows [17]:

1. The first region (Darcy law) is the Darcian flow regime at extremely low (<1 mm·s^−1^) fluid velocities—there is a linear relationship between the friction factor and *Re_i_*, and viscous forces are predominant.
2. At the upper end of the Darcian flow regime, the transition flow starts and the linear relation between the friction factor and *Re_i_* starts to bend, and the viscous forces are not predominant anymore.
3. The third region (Turbulent flow) is the Forchheimer or turbulent flow regime, where the form drag forces are dominant. The *Fanning* friction factor gets close to zero at the upper end of the transition zone, and the curve becomes almost horizontal.

An equation on the form *f = a/Re_i_ + b* (where *a* and *b* are constants) can express the equality between the *Fanning* friction factor and *Re_i_*:

(4) $$\frac{d\Delta P}{2L\rho u^{2}}=\frac{a}{Re_{i}}+b\to \frac{\Delta P}{L}=\frac{2\rho u^{2}}{d}\left(\frac{a}{Re_{i}}+b\right)\to \frac{\Delta P}{L}=\frac{2a\mu u}{d^{2}}+\frac{2b\rho u^{2}}{d}$$

This relation is just another form of the Forchheimer equation describing the fluid transport of a single-phase fluid through a porous medium when the pressure gradient changes with the average fluid velocity [20,21]:

(5) $$\frac{\Delta P}{L}=\frac{2a\mu u}{d^{2}}+\frac{2b\rho u^{2}}{d}\to \frac{\Delta P}{L}=\frac{\mu u}{k}+\beta \rho u^{2}$$

where *k* is the intrinsic permeability (m^2^) and relates to the effective surface length of the solid porous matrix, and *β* the form drag coefficient (m^−1^). The second term of Equations (4) and (5) illustrates the deviation of the pressure drop from the linearity where the drag forces become dominant over the viscous forces [22,23]. Both the permeability (*k*) and the form drag coefficient (*β*) relate the energy dissipation in a porous medium to structural characteristics such as pore geometry and porosity [17]. 

Ergun and Orning introduced a semi-empirical equation based on the structural characteristics of packed beds of spheres and adapted the Forchheimer equation to define the pressure gradient [24]:

(6) $$\frac{\Delta P}{L}=150\frac{{\left(1-\phi \right)}^{2}}{\phi^{3}}\frac{\mu u}{d_{p}^{2}}+1.75\frac{\left(1-\phi \right)}{\phi^{3}}\frac{\rho u^{2}}{d_{p}}$$

where *ϕ* is the total porosity (dimensionless) and *d_p_* the spherical particle diameter (m). The porosity is the ratio of the pore space to the bulk volume, and the pore space is either the effective or the non-effective flow through the porous medium. In view of this, the effective porosity is defined as the ratio of the interconnected (effective) pore space (*V_ev_*) to the bulk volume (*V_b_*) [17]:

(7) $$\emptyset_{e}=\frac{V_{ev}}{V_{b}}$$

**Figure 3:**
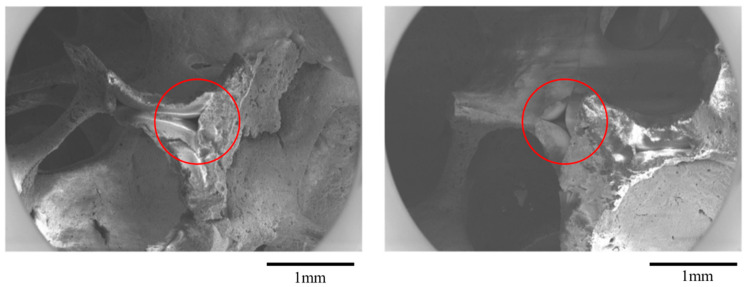
Scanning Electron Microscopy (SEM) micrograph of a triangular pore in a CFF that remained after burning out the impregnated foam.

The non-effective porosity is, however, the non-interconnected pores in the form of triangular holes in the struts that remained after burning out the impregnated foam, see Figure 3, which is the main disadvantage of the CFF manufacturing method as it diminishes the strength of the CFF [1]. 

**Figure 4:**
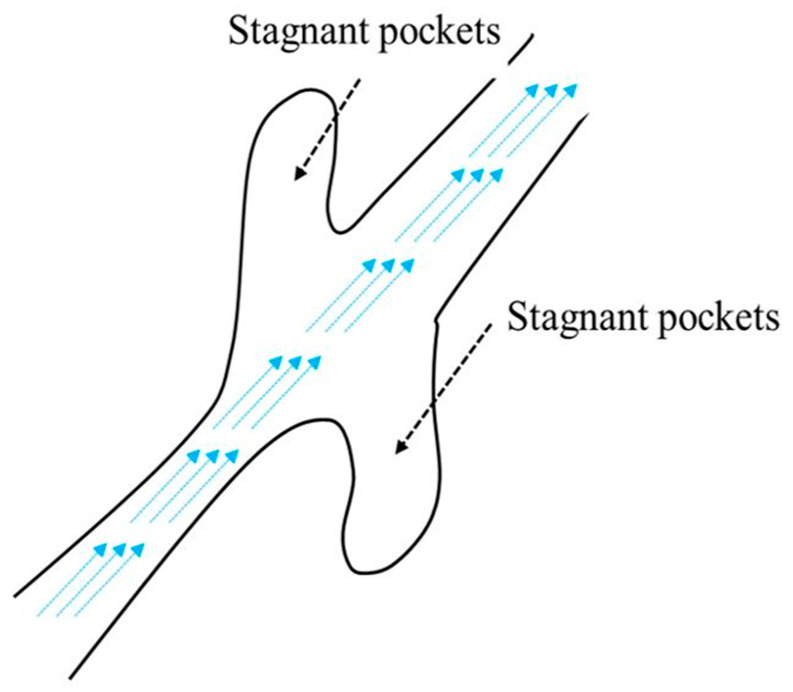
Schematic representation of stagnant pocket pores in a porous medium. Based on Ref. [17].

The dead-end pores and stagnant pockets are considered as non-effective spaces because they contribute very little to the flow in the porous medium. Figure 4 illustrates the schematic of stagnant pockets in a porous medium where the fluid is almost static in such pores [17].

The dissociation of non-effective pores from effective pores is not straightforward, and, therefore, the absolute or total porosity is often measured using Equation (8) [17]:

(8) $$\emptyset =\frac{V_{v}}{V_{b}}=\frac{\left(V_{b}-V_{s}\right)}{V_{b}}$$

where *V_v_* and *V_s_* are the voids and solid matrix volume, respectively. In this definition, *V_v_* is the total void space, regardless of whether the pores are interconnected. 

Equation (6), or a modified version, has been extensively used to evaluate the pressure drop over open foams, including CFFs [19,21,25,26]. Kennedy et al. [19], however, proposed a modified version of Ergun’s equation to determine the pressure gradient over CFF for applications in aluminum filtration:

(9) $$\frac{\Delta P}{L}=23.4\frac{\mu u}{\emptyset d_{c}^{2}}+2.00\frac{\rho u^{2}}{\emptyset^{2}d_{c}}$$

where *d_c_* is the mean *Cell* diameter for CFF of Grades 30–80 in the water velocity range of 30–800 mm·s^−1^. A deviation between the calculated pressure gradient and the experimental results of ~30% was obtained. Water was used as the fluid medium as its dynamic viscosity is comparable to molten Al at its casting temperature, i.e., ~ 1003 K (730 °C). However, since the constants of the viscous and form drag terms (23.4 and 2.00) were defined from pressure drop experiments at highly turbulent flow regimes, a more significant error would be expected when calculating the pressure drops at fluid velocities in the range of Darcy, transient, and the onset of turbulent flow regimes. 

## 3. Materials and Methods

Commercial Al_2_O_3_-based CFF of various Grades/PPI numbers were supplied from three different manufacturers, see Table 1. The name of the CFF suppliers has not been disclosed due to confidentiality agreements, and they are therefore only referred to as manufacturers A, B, and C. 

### 3.1. Sample Preparation

**Figure 5:**
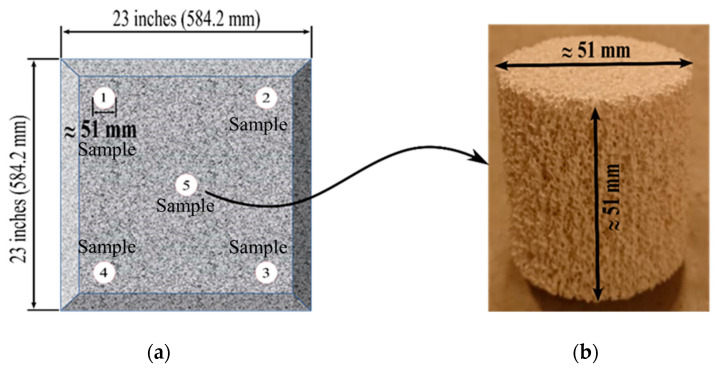
(**a**) Schematics of a Ceramic Foam Filer (CFF) block and the locations where the five samples were taken, (**b**) a cylindrical sample drilled from a CFF of Grade 50.

Three CFF blocks of every Grade/PPI numbers were used to secure the samples to be used in the pressure drop experiments. In total, five cylindrical samples were drilled from each filter block, i.e., from the center and the corners, see Figure 5a. In Figure 5b, a secured cylindrical sample from a CFF of Grade 50 is presented. 

The actual diameter and height of the samples were measured using a digital caliper from Mitutoyo, model CD-15DAX, (Kanagawa, Japan), with a resolution of 0.01 mm, and the weight using a scale from Mettler Toledo, model ME204, (Greifensee, Switzerland), with a readability of 0.1 mg and a maximum capacity of 220 g. The uncut surface of the cylindrical CFF samples was scanned using a Perfection V330 Photo Scanner from Seiko Epson Corporation, ( Tokyo, Japan), with a resolution of 2400. The captured images were used to determine the characteristic length scale of each CFF sample, i.e., the *Cell* diameter (*d_C_*) and the *Window* diameter (*d_w_*).

**Figure 6:**
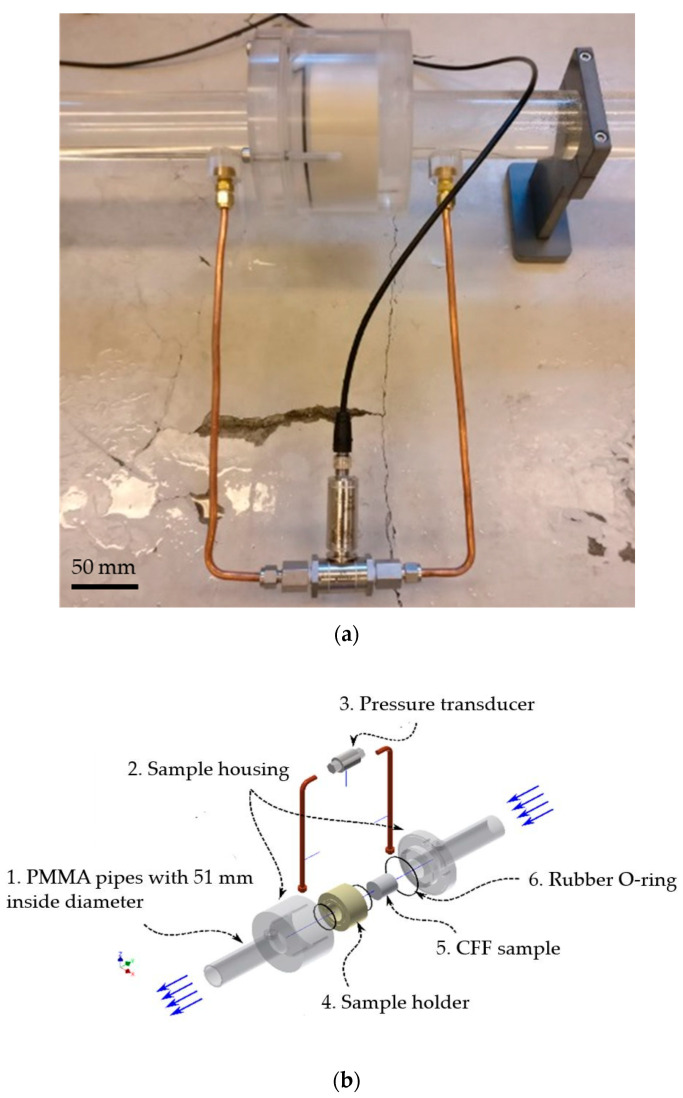
The sample housing in more detail. (**a**) setup for holding the CFF sample and measuring the pressure drop using a transducer. (**b**) The exploded 3D CAD drawing of the sample housing: (1) PMMA (Poly Methyl Methacrylate) pipes to transport the water in the system, (2) Plexiglas housing to hold the CFF sample holder in place throughout the experiment, (3) Pressure transducer, (4) sample holder encompassing the sample to make a straight-through filter design, (5) CFF sample, and (6) Rubber O-rings used to prevent water bypass between the sample holder and the housing.

A thin layer (1–2 mm) of a sealing glue (Casco SuperFix, Sika Group, Baar, Switzerland) was overlaid on the outer wall of the samples and dried at room temperatures for at least 24 h before the surplus dried glue was removed so that the sample would fit in the sample holder shown in Figure 6.

Before pushing the sample into the holder, the inner wall of the holder was covered by high-viscosity silicone grease to smoothen the surface and prevent bypassing of water. The sample holder and the interior sample were then inserted inside the housing, and the two parts were tightened using screws. Highly sensitive pressure transducer from AEP, model DF2R 100 mbar, (Modena, Italy), with a resolution of 0.001 V and ±0.03 % expanded uncertainty, were used to measure the pressure drop over the filters. The output voltage signal from the pressure transducer was data-logged using a multimeter from FLUKE, model 289 TRUE RMS, (Everett, WA, USA), with a resolution of 0.001 mV and an expanded uncertainty of 0.025% mV. 

### 3.2. Total Porosity

The total porosity of the CFF samples was calculated using Equation (8). The bulk volume (*V_b_*) was naturally determined using the dimensions of the samples, as well as the volume of the solid matrix (*V_S_*), i.e., using the sample’s weight and the true particle density of the CFF material. The particle density of the different CFF samples was obtained using the gas expansion method (Pycnometry), a standard method to measure the volume and absolute density of a porous material matrix. Accordingly, a fully automatic gas displacement Pycnometer from the Micromeritics Instrument Corporation, AccuPyc II 1340, Norcross, GA, USA, was used, and the average true density of the CFF samples measured. 

### 3.3. Physical Morphology

The mean diameter of the *Cells* and *Windows* of the CFF samples was determined using the images secured from scanning the uncut surfaces of the filters. An average area of ~4 cm^2^ was assigned to the CFF samples, and the characteristic length scales (*Cells* and *Windows*) were determined using an image analysis software, i.e., the i-Solution DT software, from IMT i-Solution Inc., (Tempe, AZ, USA). The principle of the method was based on manipulating the threshold to contrast the *Cells* or *Windows* from the rest of the geometry and thereby automatically measure the *Feret* diameter of each sample from which the mean and standard deviation could be derived. 

### 3.4. Pressure Drop Test

**Figure 7:**
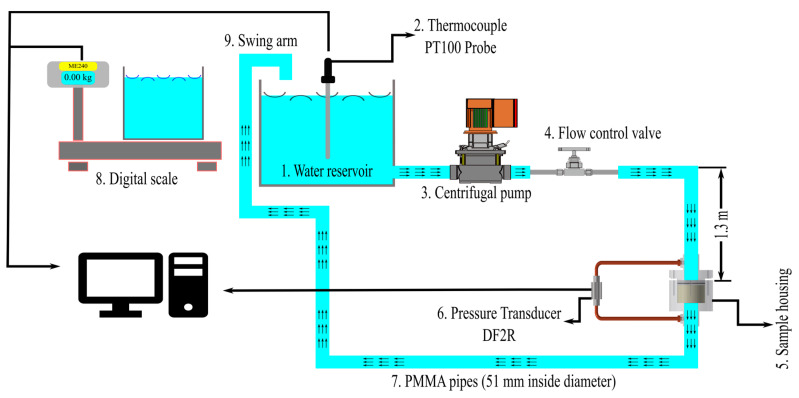
Schematic drawing of the experimental pressure drop setup.

In Figure 7, a schematic illustration of the pressure drop setup used in the present study is shown. The water reservoir with a capacity of 700 L was filled with tap water at 283 ± 3 K (10 ± 3 °C) with a viscosity of 0.0013 Pa·s which is equal to viscosity of molten aluminum at its casting temperature 993–1003 K (720–730 °C). Water is an excellent physical analogue to Al, and it can be used to study molten Al fluid dynamics.

The different parts of the setup have been numbered and are explained below:

1. The water reservoir.
2. The thermocouple Probe PT100 and data logger GMH3750 from GHM Group, Remscheid, Germany) to measure the water temperature. The thermocouple was factory-calibrated to an expanded uncertainty of ±0.22 °C with a coverage factor of 2. The software (GSOFT 3050, GHM Group, Remscheid, Germany) was used to transfer data from the logger to the PC.
3. The vertical multistage centrifugal pump (GRUNDFOS, Bjerringbro, Denmark) with integrated frequency converter, maximum 10 bar pressure and 20.5 m^3^·h^−1^.
4. The stainless-steel needle valve regulates the flow of water through the system allowing adjustment of the velocity.
5. The sample housing.
6. The differential pressure transducers DF2R.
7. Poly Methyl Methacrylate (PMMA) pipes used to transport the water in the system. The inlet pipe was 1.3 m long to secure a fully developed laminar flow in the low water velocity range. This length was calculated using Equation (10) where *D* is the pipe diameter (m), *L_E_* the entrance length (m), and *Re_D_* the Reynolds number of the pipes calculated from the defined flow rate (dimensionless), i.e., 10 mm·s^−1^,:
(10) $$\frac{L_{E}}{D}\approx 0.05Re_{D}$$
8. The digital scale model ME204, METTLER TOLEDO, Switzerland, with the readability of 0.1 mg and a maximum capacity of 220 g. The scale was equipped with a LAB-it View software (version 5.0.1), for computer data storage.
9. The swingarm that was part of the piping system.

Due to some temperature fluctuations in the experiments, the exact viscosity and density of water were calculated using the following relationships [27,28]:

(11) $$log\mu =A+\frac{B}{C-T}$$

(12) $$\begin{array}{ll} \rho_{T}=999.842594 & +6.793952\times 10^{-2}T-9.095290\times 10^{-3}T^{2}\\ & +1.001685\times 10^{-4}T^{3}-1.120083\times 10^{-6}T^{4}\\ & +6.536332\times 10^{-9}T^{5} \end{array}$$

where *T* is the temperature (K), *µ* the dynamic viscosity (kg∙m^−1^∙s^−1^), and *A*, *B*, and *C* constants with a magnitude of 4.5318, −220.57, and 149.39, respectively, in the temperature range 276 – 380 K (3 – 107 °C). The water temperature was measured and logged using the OMEGA thermocouple PT100 from GHM Group (Remscheid, Germany), connected to the control software GSOFT3050 V3.6. 

The air in the pipes and the CFF samples had to be eliminated to secure stable conditions throughout the experiments. Accordingly, a loop for the water to circulate at high pressure before entering the experimental setup was established. The loop was interrupted by turning the moving part of the piping system, i.e., the swingarm shown in Figure 7, over the container placed on the digital scale. The water accumulation was measured and logged using the digital scale. A constant flow rate was established, and the corresponding pressure drop, and accumulation rate of water were logged for 30–60 s. Ten to twenty different velocities and their corresponding pressure drop were data-logged for each CFF sample. The superficial velocity (*u*) was determined from the mass of the accumulated water, the density of the water, and the measured cross-sectional area of the CFF samples. The permeability was later determined using measured data for the pressure drop through the filter, the sample dimensions, the water viscosity, and the superficial velocity.

## 4. Results and Discussion

### 4.1. Porosity

The true particle density of the CFF samples and their average are summarized in Table 2. As can be seen from the table, the average true particle density for the CFFs from suppliers A and B were relatively similar and slightly higher than those of the CFFs from supplier C. 

The porosity of the CFF samples was calculated using Equation (8), and the average values for the different Grades/PPI numbers are summarized in Table 3 and the results for each of the individual CFF samples can be found in Table A1, Table A2, Table A3 and Table A4. As can be seen from the table, the high-Grade/PPI number CFFs (65 and 80) from suppliers A and B showed slightly lower porosity than the low-Grade/PPI number CFFs (30). However, this trend was not observed for the CFFs from supplier C. The reason for this is believed to be directly linked to the uneven cylindrical shape of the samples secured from the 50 PPI and 60 PPI CFF blocks from supplier C which later also gave an increased uncertainty in measuring the sample dimensions. Nevertheless, the obtained result clearly indicated that the porosity of the different CFF samples was somewhat similar.

### 4.2. Mean Feret Diameter of the Cell and the Window 

To better understand the differences between the CFF samples in view of their physical morphologies, the mean *Feret* diameter of the *Cells* and *Windows* of each Grade/PPI number was calculated and presented together with the pooled standard deviations and the number of counts (sample population) within each Grade/PPI number, see Table 4 (the mean *Feret* diameter of the *Cells* and *Windows* of each of the individual CFF samples can be found in Table A5, Table A6, Table A7, Table A8, Table A9, Table A10, Table A11 and Table A12). As can be seen from the table, the deviations between the measured *Feret* diameter of the *Windows* proved to be less than in the case of the *Cells*. This is believed to be due to how they were measured, i.e., the circumferences of the *Windows* were identified through thresholding with a higher degree of precision than in the case of the *Cells*. It can also be seen from the Table 4 that CFF samples of corresponding Grade/PPI number clearly have different values for the *Feret* diameter of the *Cells* and *Windows*. In some cases, the values are significantly different, e.g., the average CFF *Window* diameter of Grade 30 from supplier A was 27% less than that of the 30 PPI from supplier C. It should be noted that in the grading system, the *Cell Feret* diameter defines the range of each CFF Grade. However, as the *Windows Feret* diameter can be measured with more precision, it is believed that it can substitute the *Cell* size in the grading system. 

### 4.3. Permeability

**Figure 8:**
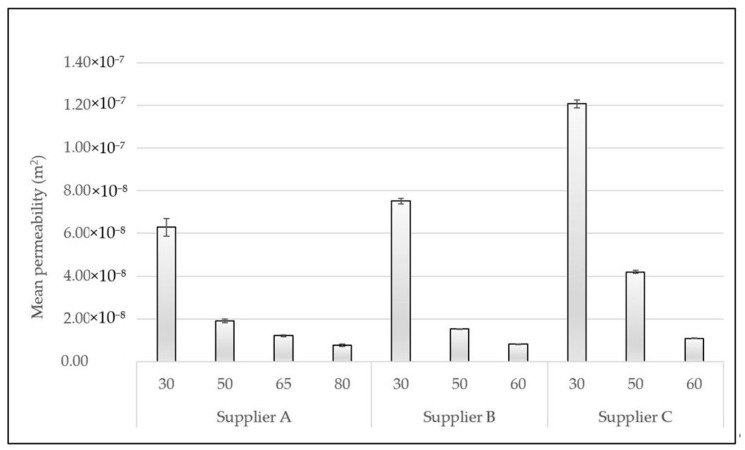
Mean permeability of CFFs of various Grades/PPI numbers from suppliers A, B, and C.

The CFF sample permeability was measured based on pressure drop experiments performed at the velocity range ≤ 10 mm·s^−1^. In Figure 8 the calculated mean permeability of all the investigated CFFs is presented. The permeability of each of the individual CFF samples within each Grade/PPI number can be found in Table A13, Table A14, Table A15 and Table A16.

As can be seen from Figure 8, the permeability of CFF 30 PPI and CFF 50 PPI from supplier C is significantly higher than the corresponding CFFs from suppliers A and B, i.e., Grade 30 and Grade 50 from supplier A, as well as 30 PPI and 50 PPI from supplier B, which, as previously mentioned, is believed to be linked to the disparity of their structural morphologies, see Table 4. This observation indicates that the permeability of the CFFs of a specific class can be relatively different in view of their structural morphologies and fluid flow properties when originating from different suppliers. 

The permeability or morphological characteristics of the samples investigated in the present study, i.e., of the CFFs within each Grade/PPI, were defined using the statistical two-sample t-test with a 95% confidence interval. Before executing the t-test, the equality of the variances between the two datasets was tested to identify what assumption, i.e., equal, or unequal variances, should be practiced for the unpaired t-test. The test was accomplished by performing a two-sample F-test with a 95% confidence interval and by assuming equal variances for each pair of the dataset, i.e., the Null hypothesis. In Table 5, the results from the statistical analysis in view of identifying the variations in the mean sample permeability of CFFs Grade 30 from supplier A and 30 PPI from supplier B are presented. As can be seen from the table, the Null hypothesis could not be rejected, concluding that the variation of the mean permeability of CFFs Grade 30 from supplier A and CFFs 30 PPI from supplier B was not statistically significant.

An identical statistical analysis was performed to identify the corresponding CFF from suppliers B and supplier C to CFF from supplier A. A summary of the results is shown in Table 6 correlating the identical CFF using colored boxes. The results in Table 6 are valid for statistical analysis of both permeability and the *Feret* diameter of the *Window* and confirm/highlight the lack of a systematic way of sorting CFFs as there are two different classification systems. No correlation can even be identified between the CFFs classified using the same system, as in the case of suppliers B and supplier C.

### 4.4. Flow Regimes in CFF

**Figure 9:**
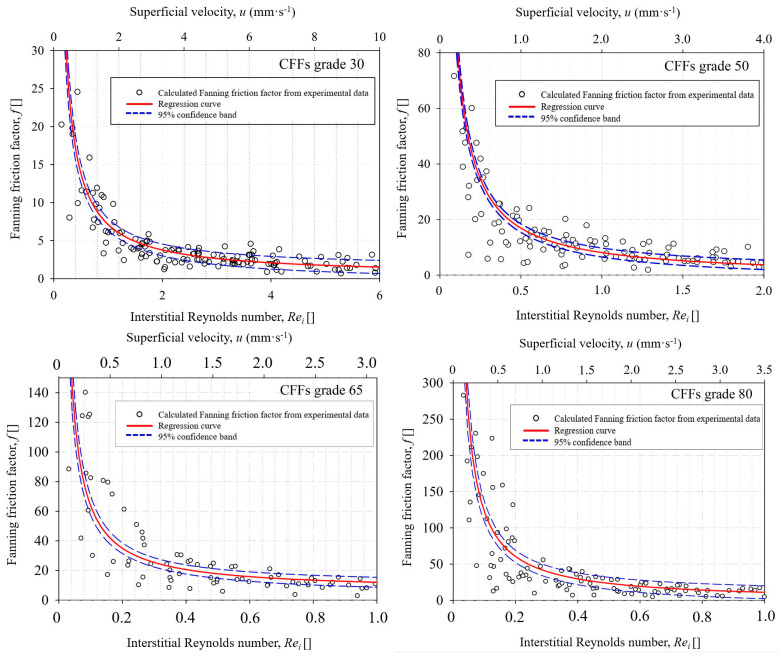
The *Fanning* friction factor (dimensionless) as a function of the interstitial Reynolds numbers (*Re_i_*) and superficial velocity (*u*) for CFFs Grade 30–80 from supplier A.

The velocity at which the laminar flow is transferred to transient flow and eventually turbulent flow can be identified using graphs correlating the dimensionless *Fanning* friction factor to *Re_i_* number, where the mean *Window* Feret diameter of CFF samples is used as the characteristic length scale for the estimation of *Re_i_* number. In Figure 9 the relation of the *Fanning* friction factor as a function of *Re_i_* number for CFF grade 30–80 from supplier A is presented. As can be seen from the Figure, the graph becomes almost horizontal at the onset of the turbulent flow regime, where the *Fanning* factor approaches zero.

The obtained laminar, transition, and turbulent flow regimes can be recognized by analogy with the three flow regions presented in Figure 2. The linear section of the diagram showing the laminar flow regime and the onset of the transitional flow regime can be defined by drawing a tangent line from the linear section of the graph to the interception with the “x” axis. In the laminar flow region, the friction factor of CFFs Grade 80 is one order of magnitude higher than the CFFs Grade 30, indicating that the wall shear stresses are higher for the CFFs with smaller pore sizes. As previously mentioned, the transitional flow region is reached a point where the drag forces are dominant, and the inertial forces are comparable to viscous forces. After that, the graph starts to decay until the velocity graph flattens horizontally. 

The flow regimes in the CFFs from suppliers B and supplier C were also evaluated, and the results indicated that a laminar flow was achieved through CFFs of Grades/PPI numbers 30–80 at *Re_i_* ≤ 1, which corresponds to a superficial fluid velocity of ≤2 mm·s^−1^. This velocity is lower than the normally applied Al filtration velocities in the DC casting process, i.e., the velocity is in the superficial velocity range at which the flow in the channels is transient or turbulent. The presently obtained results are contrary to the assumption made by Bao et al. [8], where the laminar (creeping) flow was considered in a model of particle removal in CFFs during Al filtration. In view of this, a higher filtration efficiency was obtained in the derived model compared to the experimental work as the influence of the inertia as lifting forces on the collected particles partly resulted in particle re-entrainment.

**Figure 10:**
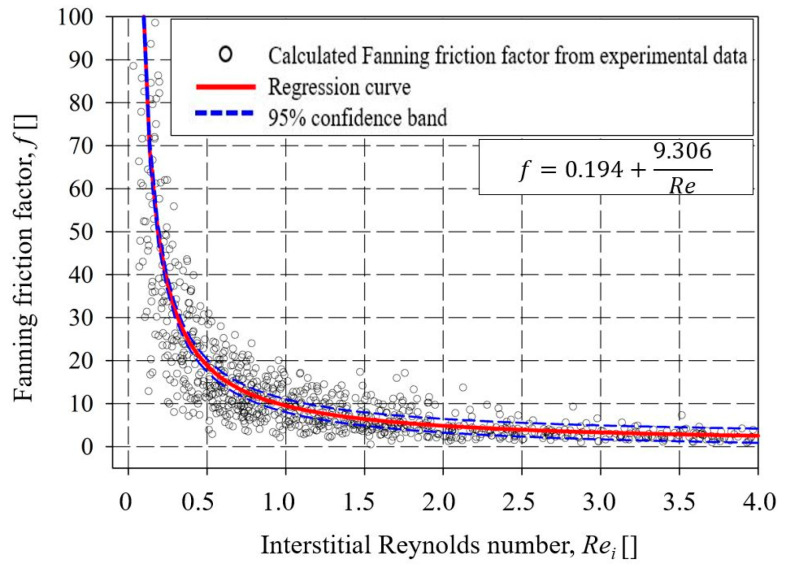
The *Fanning* friction factor as a function of interstitial Reynolds numbers (*Re_i_*) of all the data points from supplier A, B, and C.

In the present study, the obtained pressure drop variations in the investigated CFFs can be analyzed through the model equations derived from graphs of *Fanning* friction factor (*f*) versus *Re_i_* number and their correlation to the Forchheimer equation (Equation (5)). In correlation to a characteristic length scale of CFFs, the model is identical to the Ergun equation (Equation (6)) that can derive the constants *a* and *b* in Equation (4) for CFFs of a specific Grade/PPI number. However, deriving global constants is, for straightforwardness, of more interest. Accordingly, the calculated *Fanning* friction factor (*f*) of all the investigated CFFs in the present study was plotted against the corresponding *Re_i_* number, and a model equation was derived using regression analysis, see Figure 10. 

As can be seen from the figure, a nonlinear trend line at 95% confidence interval was fitted through the data points using regression analysis, and a polynomial inverse first-order equation was derived. The resulting function was later modified by substituting Equation (3) for the *Fanning* friction factor (*f*), and correlating the pressure drop of the CFFs to their characteristic length scale, i.e., to the *Window Feret* diameter (*d_w_*), see Equation (13):

(13) $$\frac{\Delta P}{L}=\frac{18.612\mu u}{d_{w}^{2}}+\frac{0.388\rho u^{2}}{d_{w}}$$

**Figure 11:**
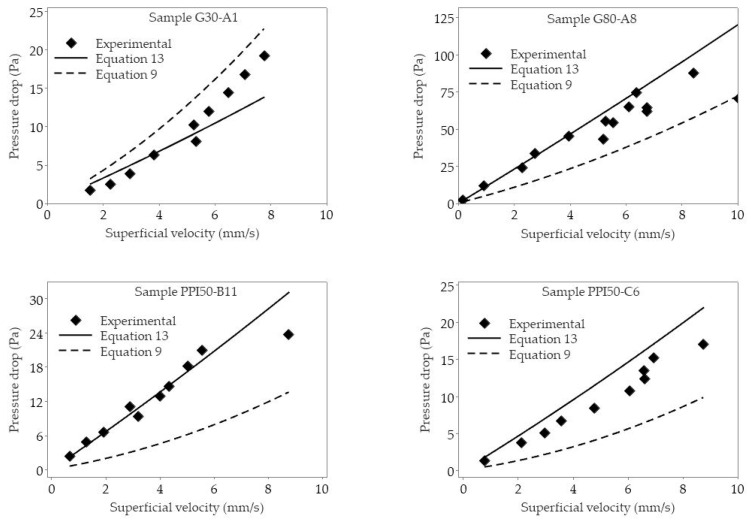
The obtained pressure drops calculated using Equations (13) and (9) (published by Kennedy et al. [10]), as well as the experimentally measured pressure drop values as a function of the superficial velocity (*u*) for CFF samples.

Equation (13), together with a previously published equation by Kennedy et al. [19] (Equation (9)), were then plotted against the experimental pressure drop values obtained for CFF samples from suppliers A, B, and C to find the model that could describes better the experimental work, see Figure 11. The *Cell Feret* diameter and *Window Feret* diameter of the samples were used, respectively, for *d_c_* and *d_w_* in Equations (9) and (13), see Table A1, Table A2, Table A5, and Table A6 for the measured data of *d_c_*, *d_w_,* and porosity of all the samples.

In Equation (9), the *Cell Feret* diameter (*d_c_*) was substituted for the equivalent particle size (*d_p_*) in the Ergun equation (Equation (6)) using Equation (14) which allowed to obtain an equivalent particle size (*d_p_*) for CFFs based on their porosity (*Ø*) and mean *Cell Feret* diameter (*d_c_*) [19]:

(14) $$d_{p}=1.5\frac{\left(1-\emptyset \right)}{\emptyset }d_{c}$$

In Figure 11, the presently measured experimental and calculated pressure drops values are shown by respective symbols and regression lines. As can be seen from the figure, there is an increased agreement between the obtained results based on the presently derived equation, i.e., Equation (13), and the experimental values when compared with the calculated pressure drop values based on the equation proposed by Kennedy et al. [10], i.e., Equation (9). It should, however, be noted that Kennedy et al. [10] used a similar experimental setup as in the present study but used a maximum velocity of 800 mm·s^−1^. In contrast, in the present case, the velocity used was set to simulate industrial casting conditions, i.e., ≤ 10 mm·s^−1^. 

In the filtration of Al using CFFs, the filters are placed inside a filter box and preheated to a temperature of about 993 K (720 °C) to prevent solidification of the molten Al metal when it initially meets the filter at the start of the filtration step [29]. As a result, a metallostatic head is formed on the surface of the CFF, and the pressure from the metal head breaks the oxides layer that has formed on the interface between the CFF surface and the molten Al, thereby primes the filter, i.e., infiltrates the CFFs with molten Al [16]. From that point, the filtration process is initiated, and the molten Al flows through the CFF [16]. Equations (9) and (13) can both be used to evaluate the required metallostatic head needed to prime the CFF, and a metal head of 21.3 cm and 8.9 cm were presently obtained. In view of this result, it is clear that pressure drop experiments must be performed at velocities close to industrial casting conditions for Al to be able to accurately study and evaluate the hydraulic properties of CFF during the Al filtration. 

## 5. Conclusions and Future Work

The present study investigates the morphological characteristics and permeability of commercial alumina (Al_2_O_3_) CFF. The main goals of the study have been to (i) define the different flow regimes inside CFFs at flow velocities in the range of industrial filtration of Al (10 mm·s^−1^) and (ii) derive a semi/empirical equation for calculation of pressure drop and the permeability of CFFs using their morphological characteristics. Based on the present investigation, the following conclusions have been made:

1. Comparing the permeability and mean *Window Feret* diameter of different filters confirmed the need for a systemic method to categorize CFFs.
2. Filtration of Al using CFF is performed at the superficial velocity at which the fluid flow in the channels is turbulent.
3. The derived empirical model for the calculation of the pressure drop of CFFs correlates well with the experimental pressure drop data.
4. Empirical expressions for calculating the pressure drop over CFF should be derived based on experimental measurements carried out at the velocity range of the application.

For the future work, the structural uniformity of CFFs and the effect of different flow regimes on the permeability of the filters will be investigated using pressure drop experiments at high and low fluid velocities.

## Figures and Tables

**Table 1:** The dimensions and Grade/PPI numbers of the CFF used in the present study.

CFF Supplier	Dimensions (Length × Width × Height) (mm)	Grade	PPI
A	584.2 × 584.2 × 51	30, 50, 65 and 80	-
B	584.2 × 584.2 × 51	-	30, 50, and 60
C	508 × 508 × 50	-	30, 50, and 60

**Table 2:** The true particle density of CFFs from suppliers A, B, and C.

CFF Supplier		True Particle Density (kg·m^−3^)	Std. Dev. (kg·m^−3^)	Average True Particle Density (kg·m^−3^)
A	Grade 30	3413.6	9	3426.3
	Grade 50	3431.5	3.8	
	Grade 65	3434.4	6.2	
	Grade 80	3425.8	4	
B	30 PPI	3427.8	7.8	3427.9
	50 PPI	3417.3	8.3	
	60 PPI	3438.8	4.4	
C	30 PPI	3398.3	8.1	3388.8
	50 PPI	3391.7	7.3	
	60 PPI	3376.5	3	

**Table 3:** The average porosity of CFF from suppliers A, B, and C.

Supplier A				Supplier B			Supplier C		
G * 30	G 50	G 65	G 80	30 PPI	50 PPI	60 PPI	30 PPI	50 PPI	60 PPI
0.88	0.85	0.86	0.85	0.88	0.87	0.87	0.88	0.89	0.89
* Grade									

**Table 4:** The mean *Feret* diameter of the *Cell* and *Window* of CFF from suppliers A, B, and C.

Supplier and Grade/PPI Number	Counts (Number of Samples)	*Cell*		*Window*	
		Mean *Feret* Diameter (µm)	Pooled Std. Dev. (µm)	Mean *Feret* Diameter (µm)	Pooled Std. Dev. (µm)
A Grade 30	12	1254.7	893.1	751.0	262.9
A Grade 50	14	1047.8	577.8	503.5	388.6
A Grade 65	12	886.3	531.4	407.2	109.3
A Grade 80	12	655.9	460.2	345.8	102.2
B 30 PPI	10	1323.5	872.0	697.2	172.8
B 50 PPI	15	1134.1	722.6	501.0	93.7
B 60 PPI	14	881.1	568.3	354.4	69.7
C 30 PPI	14	2134.1	1219.1	960.3	249.1
C 50 PPI	15	1429.3	793.5	725.2	167.7
C 60 PPI	13	900.4	535.3	389.3	73.4

**Table 5:** The descriptive statistics of the F-Test, examining the equality of the variances of the permeabilities of the CFF samples and subsequently testing the significance of the variation of their mean permeability using the *t*-test. The CFFs tested were Grade 30 from supplier A and 30 PPI from supplier B.

	Equality of the Sample Variances (F-Test)		Significance of the Means (*t*-Test, Equal Variances)	
Mean Permeability (m^2^)	A30 = 6.29 × 10^−8^	B30 = 7.5 × 10^−8^	A30 = 6.29 × 10^−8^	B30 = 7.5 × 10^−8^
Observation Number	A30 = 14	B30 = 9	A30 = 14	B30 = 9
Degrees of Freedom (d_f_)	A30 = 13	B30 = 8	21	
F or T-critical (One tail)	0.36		1.72	
Test Statistic	0.28		0.82	
Null (H0)	S *_A30_^2^ = S_B30_^2^		$\hat{\mu }{\;}^{**}{\;}_{A30}={\hat{\mu }}_{B30}$	
Reject Null	No		No	
* “S” is the sample standard deviation. ** “ μ ” is the sample mean.				

**Table 6:** The corresponding CFF of 30, 50, and 60 PPI from supplier B and supplier C with the Grades 30, 50, 65, and 80 from supplier A.

		Supplier B			Supplier C		
		PPI 30	PPI 50	PPI 60	PPI 30	PPI 50	PPI 60
Supplier A	Grade 30						
	Grade 50						
	Grade 65						
	Grade 80						

## Data Availability

All the data created are presented in the Appendix A.

## Appendix A

**Table A1:** The porosity of CFF samples of Grade 30 and 50 from supplier A.

	Grade 30		Grade 50	
	Porosity	Uncertainty	Porosity	Uncertainty
A1	8.72 × 10^−1^	3.92 × 10^−4^	8.39 × 10^−1^	8.19 × 10^−4^
A2	8.79 × 10^−1^	7.30 × 10^−4^	8.50 × 10^−1^	1.09 × 10^−3^
A3	8.98 × 10^−1^	8.09 × 10^−4^	8.68 × 10^−1^	1.22 × 10^−3^
A4	8.77 × 10^−1^	1.02 × 10^−4^	8.41 × 10^−1^	1.16 × 10^−3^
A5	8.84 × 10^−1^	9.95 × 10^−4^	8.50 × 10^−1^	7.68 × 10^−4^
A6	8.69 × 10^−1^	6.36 × 10^−4^	8.49 × 10^−1^	8.47 × 10^−4^
A7	8.80 × 10^−1^	8.87 × 10^−4^	8.56 × 10^−1^	2.43 × 10^−3^
A8	8.94 × 10^−1^	8.37 × 10^−4^	8.42 × 10^−1^	8.42 × 10^−4^
A9	8.74 × 10^−1^	1.32 × 10^−4^	8.33 × 10^−1^	1.05 × 10^−3^
A10	8.83 × 10^−1^	7.53 × 10^−4^	8.45 × 10^−1^	9.21 × 10^−4^
A11	8.98 × 10^−1^	1.64 × 10^−4^	8.41 × 10^−1^	7.33 × 10^−4^
A12	8.95 × 10^−1^	4.98 × 10^−4^	8.43 × 10^−1^	1.19 × 10^−3^
A13	8.95 × 10^−1^	1.54 × 10^−4^	8.60 × 10^−1^	1.14 × 10^−3^
A14	8.98 × 10^−1^	1.01 × 10^−4^	8.48 × 10^−1^	1.14 × 10^−3^
A15	8.75 × 10^−1^	6.79 × 10^−4^	8.47 × 10^−1^	1.37 × 10^−3^

**Table A2:** The porosity of CFF samples of Grade 65 and Grade 80 from supplier A.

	Grade 65		Grade 80	
	Porosity	Uncertainty	Porosity	Uncertainty
A1	8.73 × 10^−1^	9.35 × 10^−4^	8.63 × 10^−1^	1.11 × 10^−3^
A2	8.54 × 10^−1^	1.00 × 10^−3^	8.73 × 10^−1^	3.97 × 10^−3^
A3	8.47 × 10^−1^	9.33 × 10^−4^	8.55 × 10^−1^	4.79 × 10^−3^
A4	8.49 × 10^−1^	7.50 × 10^−4^	8.51 × 10^−1^	1.47 × 10^−3^
A5	8.54 × 10^−1^	1.00 × 10^−3^	8.58 × 10^−1^	2.26 × 10^−3^
A6	8.43 × 10^−1^	1.12 × 10^−3^	-	-
A7	8.42 × 10^−1^	1.41 × 10^−3^	8.63 × 10^−1^	2.72 × 10^−3^
A8	8.45 × 10^−1^	1.19 × 10^−3^	8.44 × 10^−1^	3.12 × 10^−3^
A9	8.77 × 10^−1^	1.89 × 10^−3^	8.40 × 10^−1^	1.75 × 10^−3^
A10	8.58 × 10^−1^	2.01 × 10^−3^	8.57 × 10^−1^	1.38 × 10^−3^
A11	8.47 × 10^−1^	9.59 × 10^−4^	8.55 × 10^−1^	2.63 × 10^−3^
A12	8.46 × 10^−1^	1.82 × 10^−3^	8.58 × 10^−1^	2.42 × 10^−3^
A13	8.76 × 10^−1^	1.67 × 10^−3^	8.59 × 10^−1^	2.02 × 10^−3^
A14	8.55 × 10^−1^	1.34 × 10^−3^	8.42 × 10^−1^	1.76 × 10^−3^
A15	8.60 × 10^−1^	1.24 × 10^−3^	8.49 × 10^−1^	1.02 × 10^−2^

**Table A3:** The porosity of CFF samples of 30 PPI, 50 PPI and 60 PPI from supplier B.

	30 PPI		50 PPI		60 PPI	
	Porosity	Uncertainty	Porosity	Uncertainty	Porosity	Uncertainty
B1	8.83 × 10^−1^	2.89 × 10^−4^	-	-	-	-
B2	8.72 × 10^−1^	3.16 × 10^−4^	-	-	-	-
B3	8.84 × 10^−1^	2.88 × 10^−4^	8.76 × 10^−1^	3.06 × 10^−4^	8.79 × 10^−1^	2.98 × 10^−4^
B4	-	-	8.69 × 10^−1^	3.24 × 10^−4^	8.82 × 10^−1^	2.91 × 10^−4^
B5	8.82 × 10^−1^	2.91 × 10^−4^	8.67 × 10^−1^	3.28 × 10^−4^	8.70 × 10^−1^	3.20 × 10^−4^
B6	8.87 × 10^−1^	2.80 × 10^−4^	-	-	-	-
B7	8.89 × 10^−1^	2.75 × 10^−4^	8.71 × 10^−1^	3.19 × 10^−4^	8.74 × 10^−1^	3.12 × 10^−4^
B8	8.77 × 10^−1^	3.04 × 10^−4^	8.80 × 10^−1^	2.96 × 10^−4^	8.79 × 10^−1^	2.98 × 10^−4^
B9	8.77 × 10^−1^	3.04 × 10^−4^	-	-	-	-
B10	8.77 × 10^−1^	3.04 × 10^−4^	8.73 × 10^−1^	1.78 × 10^−3^	8.67 × 10^−1^	3.28 × 10^−4^
B11	-	-	8.69 × 10^−1^	3.23 × 10^−4^	-	-
B12	-	-	-	-	-	-
B13	-	-	8.79 × 10^−1^	3.00 × 10^−4^	8.67 × 10^−1^	3.29 × 10^−4^
B14	-	-	-	-	8.73 × 10^−1^	3.14 × 10^−4^
B15	-	-	8.70 × 10^−1^	3.22 × 10^−4^	8.70 × 10^−1^	3.22 × 10^−4^

**Table A4:** The porosity of CFF samples of 30 PPI, 50 PPI and 60 PPI from supplier C.

	30 PPI		50 PPI		60 PPI	
	Porosity	Uncertainty	Porosity	Uncertainty	Porosity	Uncertainty
C1	8.88 × 10^−1^	2.30 × 10^−4^	-	-	8.89 × 10^−1^	2.30 × 10^−4^
C2	8.81 × 10^−1^	2.47 × 10^−4^	8.98 × 10^−1^	2.10 × 10^−4^	8.91 × 10^−1^	2.25 × 10^−4^
C3	8.82 × 10^−1^	2.43 × 10^−4^	8.93 × 10^−1^	2.22 × 10^−4^	-	-
C4	8.86 × 10^−1^	2.37 × 10^−4^	-	-	-	-
C5	-	-	8.91 × 10^−1^	2.25 × 10^−4^	8.85 × 10^−1^	2.39 × 10^−4^
C6	8.81 × 10^−1^	2.45 × 10^−4^	8.93 × 10^−1^	2.22 × 10^−4^	8.89 × 10^−1^	2.29 × 10^−4^
C7	8.85 × 10^−1^	2.38 × 10^−4^	-	-	-	-
C8	-	-	-	-	-	-
C9	8.87 × 10^−1^	2.34 × 10^−4^	8.92 × 10^−1^	2.24 × 10^−4^	8.87 × 10^−1^	2.33 × 10^−4^
C10	8.78 × 10^−1^	2.52 × 10^−4^	8.88 × 10^−1^	2.32 × 10^−4^	8.81 × 10^−1^	2.48 × 10^−4^
C11	-	-	8.99 × 10^−1^	2.09 × 10^−4^	-	-
C12	-	-	9.03 × 10^−1^	2.00 × 10^−4^	-	-
C13	-	-	-	-	8.88 × 10^−1^	2.34 × 10^−4^
C14	-	-	-	-	8.84 × 10^−1^	2.39 × 10^−4^
C15	-	-	8.86 × 10^−1^	2.36 × 10^−4^	8.78 × 10^−1^	2.54 × 10^−4^

**Table A5:** Mean *Window’s Feret* diameter (*d_w_*) of CFF samples of Grade 30 and Grade 50 from supplier A.

	G30			G50		
	Counts	St. Dev. (µm)	Mean (µm)	Counts	St. Dev. (µm)	Mean (µm)
A1	97	252.3	853.3	240	201.1	444
A2	543	257.9	685.4	183	163.1	579.7
A3	485	256.1	654.5	485	-	386.2
A4	209	226.6	960.4	191	206.9	604
A5	105	270.4	768.9	-	-	-
A6	212	284.2	948.6	219	214.9	433.3
A7	117	254.4	759.8	158	151.3	537.1
A8	135	206.1	564.5	135	130.7	471.2
A9	83	268.2	626.5	766	173.9	464.5
A10	-	-	-	136	109.7	430.8
A11	-	-	-	146	124.4	521.7
A12	108	296.8	725.6	178	121.4	539.7
A13	-	-	-	129	147.4	573.4
A14	172	252.2	759.1	189	184	529.1
A15	446	292.3	705.8	174	133.8	534.2

**Table A6:** Mean *Window’s Feret* diameter (*d_w_*) of CFF samples of Grade 65 and Grade 80 from supplier A.

	Grade 65			Grade 80		
	Counts	St. Dev. (µm)	Mean (µm)	Counts	St. Dev. (µm)	Mean (µm)
A1	167	131.2	436.4	296	92.2	323.1
A2	106	108	398.8	271	98.9	285.1
A3	115	87.2	443.4	-	-	-
A4	217	165.7	411.1	143	78.5	283.3
A5	-	-	-	94	67.3	305.3
A6	169	67.8	456	200	147.1	427.9
A7	168	120.9	416.1	101	95	397.2
A8	131	97	412.2	68	89.6	319.8
A9	195	109.3	433.3	437	77.5	287.5
A10	-	-	-	-	-	-
A11	129	93.5	437	186	86.7	363.5
A12	196	97.8	425.7	230	110.4	308.7
A13	-	-	-	231	149.1	450.3
A14	119	62.6	237.4	121	70.2	397.6
A15	170	85.7	378.6	-	-	-

**Table A7:** Mean *Window’s Feret* diameter (*d_w_*) of CFF samples of 30 PPI, 50 PPI and 60 PPI from supplier B.

	30 PPI			50 PPI			60 PPI		
	Counts	St. Dev. (µm)	Mean (µm)	Counts	St. Dev. (µm)	Mean (µm)	Counts	St. Dev. (µm)	Mean (µm)
B1	158	129	556.4	159	96.1	507.2	137	56.5	284.7
B2	127	190.5	733.6	78	74	480	140	77.8	423.2
B3	111	173.6	664.4	112	82.8	442.8	-	-	-
B4	100	136.4	636.3	-	119.1	591.2	117	81.9	370.9
B5	103	183	763.6	118	74.2	479.1	117	87	413.5
B6	131	149.2	609.8	116	86.9	469	115	59.3	331.4
B7	124	201.8	738.8	111	105.9	519.2	153	62.1	337.8
B8	127	203.5	824.8	144	102.9	563.1	124	90.3	391.5
B9	122	185	716.2	133	107.1	565	139	72.6	392.1
B10	138	165	728.5	151	88.6	461.3	92	106.5	417
B11	-	-	-	115	107.2	580.8	182	41.6	267.2
B12	-	-	-	115	93.2	521.2	-	71.4	388.4
B13	-	-	-	146	100.7	482.4	174	71.6	391.4
B14	-	-	-	174	89	444.6	116	57.9	337.1
B15	-	-	-	133	86	498.7	179	43.7	249.7

**Table A8:** Mean *Window’s Feret* diameter (*d_w_*) of CFF samples of 30 PPI, 50 PPI and 60 PPI from supplier C.

	30 PPI			50 PPI			60 PPI		
	Counts	St. Dev. (µm)	Mean (µm)	Counts	St. Dev. (µm)	Mean (µm)	Counts	St. Dev. (µm)	Mean (µm)
C1	119	250.7	991	150	176.3	775.7	374	82.5	450.8
C2	-	-	-	167	176.3	657.5	-	-	-
C3	109	229	846.2	143	192.9	789.7	210	75.4	384.2
C4	132	218.8	779.3	174	152.7	701.8	201	83.9	435.1
C5	114	226.9	928.8	129	166.3	716.5	-	80.7	436.9
C6	123	260.7	1026.8	216	163.5	703.7	232	84.8	449.2
C7	110	293.3	1044.7	160	169.3	731.3	129	64.1	370
C8	111	253.3	926	151	188.3	825.4	206	60.5	410.3
C9	148	240.8	1083.2	165	130.7	640.2	231	79.6	365.7
C10	148	218	870.5	176	162.4	712.4	201	60	332.7
C11	157	267.2	1065.4	198	158.5	696.1	174	71.2	376.8
C12	115	288.3	1040.6	133	169.4	895.8	192	79.1	410.8
C13	131	269.4	999.9	107	176.9	720.6	183	63.9	359
C14	142	259.8	995.9	150	146.2	644.2	238	59.2	334.6
C15	160	210.2	845.9	127	190.8	667.3	173	68.1	382.1

**Table A9:** Mean *Cell’s Feret* diameter (*d_C_*) of CFF samples of Grade 30 and Grade 50 from supplier A.

	Grade 30			Grade 50		
	Counts	St. Dev. (µm)	Mean (µm)	Counts	St. Dev. (µm)	Mean (µm)
A1	110	392.5	948.2	155	459.1	972.7
A2	348	1237.7	1836.5	149	771.3	1186.3
A3	561	755.7	1070.7	1203	336.2	565.3
A4	94	290.6	740.1	143	797.5	1370.7
A5	134	579.2	1179.4	109	722.6	1272
A6	162	345.3	1083	164	469.5	879.1
A7	143	630.6	1178.3	427	635.9	957.6
A8	180	411.2	827.5	178	754.7	932.8
A9	90	802.4	1661.8	948	686.9	993.4
A10	-	-	-	69	599.3	1376.1
A11	-	-	-	125	732.4	1571.5
A12	107	1624	1871.9	160	537.8	1129.6
A13	-	-	-	156	649.7	1006.9
A14	148	360.5	983.1	225	537.5	796.9
A15	421	1195.5	1676.2	97	277.4	706.4

**Table A10:** Mean *Cell’s Feret* diameter (*d_C_*) of CFF samples of Grade 65 and Grade 80 from supplier A.

	Grade 65			Grade 80		
	Counts	St. Dev. (µm)	Mean (µm)	Counts	St. Dev. (µm)	Mean (µm)
A1	240	412.4	729.2	-	-	-
A2	137	488.8	868.2	402	603.6	770
A3	62	620.6	1154.6	-	-	-
A4	219	612.2	1133.6	239	354.3	525.3
A5	-	-	-	181	435.1	713.6
A6	222	476.6	943.2	406	595.5	814.7
A7	140	444	990.9	205	474.4	759.9
A8	220	568.7	833.8	194	383.5	600.2
A9	232	452.7	720.3	682	401.3	596.2
A10	-	-	-	-	-	-
A11	158	443.7	914.9	552	527.4	342.3
A12	198	687.4	1020.8	329	282.9	594.9
A13	-	-	-	226	343	767.9
A14	186	548.9	812.4	195	310.5	739.3
A15	509	552.7	513.3	-	-	-

**Table A11:** Mean *Cell´s Feret* diameter (*d_C_*) of CFF samples of 30 PPI, 50 PPI and 60 PPI from supplier B.

	30 PPI			50 PPI			60 PPI		
	Counts	St. Dev. (µm)	Mean (µm)	Counts	St. Dev. (µm)	Mean (µm)	Counts	St. Dev. (µm)	Mean (µm)
B1	196	980.1	1259.5	189	734.2	1252.2	257	807.9	1244.2
B2	146	678.2	1491.6	135	440.2	1046	281	444.7	878.8
B3	253	1157.4	1397.6	313	893.8	1208.9	-	-	-
B4	277	1054.5	1520.6	205	505.7	1066.2	265	528.7	942.2
B5	83	491.1	1280.1	216	468.5	1003.4	336	537.9	946.3
B6	264	881	1105.2	167	744.9	1208.2	244	505.2	919
B7	218	545.5	1140.6	151	462.5	1202.8	258	566.7	835.4
B8	166	844.5	1382.5	409	567.9	919.3	252	629.2	1016.6
B9	88	709.3	1484.6	146	595.7	1216.7	175	575.9	1139.6
B10	182	613.4	1173.1	237	507.6	918.2	235	824.8	911.1
B11	-	-	-	444	1162.7	1435.3	275	548.8	846.7
B12	-	-	-	206	484.3	1012.3	247	414.1	631.7
B13	-	-	-	141	684.9	1229.8	356	583	721.8
B14	-	-	-	178	709.4	1156.8	392	346.5	559.7
B15	-	-	-	205	669.8	1135.4	365	576.9	742

**Table A12:** Mean *Cell’s Feret* diameter (*d_C_*) of CFF samples of 30 PPI, 50 PPI and 60 PPI from supplier C.

	30 PPI			50 PPI			60 PPI		
	Counts	St. Dev. (µm)	Mean (µm)	Counts	St. Dev. (µm)	Mean (µm)	Counts	St. Dev. (µm)	Mean (µm)
C1	125	1174.6	1778.9	164	991.1	1953.7	449	606.2	923.8
C2	-	-	-	274	657	1167.2	-	-	-
C3	136	1198.9	1955.1	153	1294.8	1908	236	567.4	1018.4
C4	150	1428	2017.6	192	879.8	1499.9	298	509.9	960
C5	82	1738.4	2530.5	173	876.9	1545	342	415.7	711.6
C6	75	1251.1	2342.7	155	1003.1	1770.7	429	660.5	1012.6
C7	101	1158.4	2292.3	269	515.5	1066.1	233	561	906.4
C8	99	1066.9	2030.5	264	646.3	1098.3	295	563.4	933.9
C9	109	1348.2	2211.3	354	582.2	1072.4	323	536.1	885.6
C10	123	942.1	1866.3	263	821.4	1278.3	307	465.5	835
C11	118	928.1	2011.2	195	755.3	1386.8	290	454.5	820.9
C12	102	1054.4	2147.1	282	744.3	1229.6		308	650.1
C13	92	1518.3	2582.7	129	815.3	1453.6	407	480.7	846.7
C14	112	709.8	1872	133	750.4	1396.7	301	460.1	838.1
C15	110	1355.4	2239.4	111	842.4	1614.1	244	577.8	1012.3

**Table A13:** Permeability of CFF samples of Grade 30 and Grade 50 from supplier A.

	Grade 30		Grade 50	
	Permeability (m^2^)	St. Dev. (m^2^)	Permeability (m^2^)	St. Dev. (m^2^)
1	8.80 × 10^−8^	6.70 × 10^−10^	1.00 × 10^−8^	8.60 × 10^−11^
2	4.40 × 10^−8^	6.40 × 10^−9^	2.30 × 10^−8^	3.60 × 10^−10^
3	2.80 × 10^−8^	3.30 × 10^−9^	2.40 × 10^−8^	3.80 × 10^−10^
4	4.90 × 10^−8^	1.10 × 10^−8^	**4.50 × 10^−8^**	**1.20 × 10^−9^**
5	4.10 × 10^−8^	5.00 × 10^−10^	2.50 × 10^−8^	1.20 × 10^−9^
6	7.20 × 10^−8^	7.60 × 10^−10^	-	-
7	7.40 × 10^−8^	5.10 × 10^−9^	2.50 × 10^−8^	8.60 × 10^−10^
8	3.90 × 10^−8^	1.10 × 10^−9^	1.30 × 10^−8^	5.50 × 10^−10^
9	* **2.5 × 10^−7^**	**1.10 × 10^−8^**	1.60 × 10^−8^	8.90 × 10^−11^
10	6.90 × 10^−8^	2.80 × 10^−9^	1.90 × 10^−8^	1.40 × 10^−9^
11	3.80 × 10^−8^	3.60 × 10^−10^	**7.60 × 10^−8^**	**3.90 × 10^−10^**
12	7.30 × 10^−8^	1.10 × 10^−9^	1.30 × 10^−8^	4.30 × 10^−10^
13	1.10 × 10^−7^	2.60 × 10^−9^	2.40 × 10^−8^	1.80 × 10^−10^
14	5.60 × 10^−8^	3.20 × 10^−10^	1.70 × 10^−8^	1.40 × 10^−9^
15	9.80 × 10^−8^	3.60 × 10^−9^	2.00 × 10^−8^	1.20 × 10^−9^

* The bold numbers are outliers and were disregarded in the calculations.

**Table A14:** Permeability of CFF samples of Grade 65 and 80 from supplier A.

	Grade 65		Grade 80	
	Permeability (m^2^)	St. Dev. (m^2^)	Permeability (m^2^)	St. Dev. (m^2^)
1	-	-	1.50 × 10^−8^	1.40 × 10^−9^
2	9.90 × 10^−9^	9.60 × 10^−11^	8.90 × 10^−9^	3.60 × 10^−11^
3	8.30 × 10^−9^	5.60 × 10^−10^	3.30 × 10^−9^	2.50 × 10^−11^
4	1.60 × 10^−8^	7.70 × 10^−10^	1.10 × 10^−8^	5.70 × 10^−10^
5	2.10 × 10^−8^	7.70 × 10^−10^	1.20 × 10^−8^	7.40 × 10^−11^
6	-	-	-	-
7	1.10 × 10^−8^	1.10 × 10^−10^	6.80 × 10^−9^	2.50 × 10^−11^
8	8.50 × 10^−9^	5.60 × 10^−10^	5.30 × 10^−9^	2.60 × 10^−11^
9	2.40 × 10^−8^	3.20 × 10^−10^	5.40 × 10^−9^	1.70 × 10^−11^
10	7.50 × 10^−9^	4.80 × 10^−10^	4.90 × 10^−9^	2.70 × 10^−10^
11	-	-	2.40 × 10^−9^	4.20 × 10^−10^
12	9.10 × 10^−9^	1.20 × 10^−10^	5.70 × 10^−9^	2.60 × 10^−10^
13	9.00 × 10^−9^	2.20 × 10^−10^	9.30 × 10^−9^	8.20 × 10^−10^
14	9.90 × 10^−9^	1.90 × 10^−10^	4.90 × 10^−9^	4.30 × 10^−11^
15	1.10 × 10^−8^	1.20 × 10^−10^	1.10 × 10^−8^	6.60 × 10^−10^

**Table A15:** Permeability of CFF samples of 30 PPI, 50 PPI and 60 PPI from supplier B.

	30 PPI		50 PPI		60 PPI	
	Permeability (m^2^)	St. Dev. (m^2^)	Permeability (m^2^)	St. Dev. (m^2^)	Permeability (m^2^)	St. Dev. (m^2^)
1	4.1 × 10^−8^	1.1 × 10^−9^	-	-	-	-
2	3.5 × 10^−8^	8.2 × 10^−10^	-	-	-	-
3	1.7 × 10^−7^	2.9 × 10^−9^	1.0 × 10^−8^	8.7 × 10^−11^	1.1001 × 10^−8^	2 × 10^−10^
4	-	-	1.8 × 10^−8^	1.4 × 10^−10^	9.4303 × 10^−9^	1 × 10^−10^
5	9.1 × 10^−8^	8.9 × 10^−10^	2.2 × 10^−8^	2.2 × 10^−10^	5.8 × 10^−9^	8 × 10^−11^
6	4.5 × 10^−8^	3.7 × 10^−10^	-	-	-	-
7	1.1 × 10^−7^	9.7 × 10^−10^	1.6 × 10^−8^	2.4 × 10^−10^	1.09 × 10^−8^	1 × 10^−10^
8	5.9 × 10^−8^	8.3 × 10^−10^	1.3 × 10^−8^	1.3 × 10^−10^	9.5434 × 10^−9^	9 × 10^−11^
9	1.1 × 10^−7^	1.5 × 10^−9^	-	-	-	-
10	2.1 × 10^−8^	2.2 × 10^−10^	1.5 × 10^−8^	1.3 × 10^−10^	8.45 × 10^−9^	6 × 10^−11^
11	-	-	1.6 × 10^−8^	1.8 × 10^−10^	-	-
12	-	-	-	-	-	-
13	-	-	* **5.5 × 10^−8^**	**3.1 × 10^−10^**	6.16 × 10^−9^	4 × 10^−11^
14	-	-	-	-	6.06 × 10^−9^	2 × 10^−11^
15	-	-	1.3 × 10^−8^	1.0 × 10^−10^	6.66 × 10^−9^	4 × 10^−11^

* The bold numbers are outliers and were disregarded in the calculations.

**Table A16:** Permeability of CFF samples of 30 PPI, 50 PPI and 60 PPI from supplier C.

	30 PPI		50 PPI		60 PPI	
	Permeability (m^2^)	St. Dev. (m^2^)	Permeability (m^2^)	St. Dev. (m^2^)	Permeability (m^2^)	St. Dev. (m^2^)
1	1.8 × 10^−7^	2.9 × 10^−9^	-	-	9.1 × 10^−9^	1.9 × 10^−10^
2	6.6 × 10^−8^	2.5 × 10^−9^	7.0 × 10^−8^	7.1 × 10^−10^	1.2 × 10^−8^	1.8 × 10^−10^
3	1.5 × 10^−7^	1.6 × 10^−9^	2.7 × 10^−8^	2.1 × 10^−10^	-	-
4	9.2 × 10^−8^	1.4 × 10^−9^	-	-	-	-
5	-	-	* **9.8 × 10^−8^**	**2.2 × 10^−9^**	1.2 × 10^−8^	1.7 × 10^−10^
6	8.8 × 10^−8^	8.6 × 10^−10^	3.8 × 10^−8^	5.4 × 10^−10^	9.2 × 10^−9^	1.1 × 10^−10^
7	2.2 × 10^−7^	2.4 × 10^−9^	-	-	-	-
8	-	-	-	-	-	-
9	7.0 × 10^−8^	1.1 × 10^−9^	3.8 × 10^−8^	5.5 × 10^−10^	1.1 × 10^−8^	1.1 × 10^−10^
10	9.9 × 10^−8^	6.5 × 10^−10^	4.3 × 10^−8^	7.4 × 10^−10^	1.2 × 10^−8^	4.1 × 10^−10^
11	-	-	3.9 × 10^−8^	3.3 × 10^−10^	-	-
12	-	-	4.8 × 10^−8^	1.4 × 10^−9^	-	-
13	-	-	-	-	**1.7 × 10^−8^**	**4.5 × 10^−10^**
14	-	-	-	-	1.0 × 10^−8^	6.8 × 10^−11^
15	-	-	3.2 × 10^−8^	5.0 × 10^−10^	1.2 × 10^−8^	8.7 × 10^−11^

* The bold numbers are outliers and were disregarded in the calculations.
