# Correction: Hassanabadi et al. Hydraulic Characterization of Ceramic Foam Filters Used in Aluminum Filtration. *Materials* 2023, *16*, 2805

**DOI:** 10.3390/ma17020459

**Published:** 2024-01-18

**Authors:** Massoud Hassanabadi, Thomas Berto, Shahid Akhtar, Ragnhild E. Aune

**Affiliations:** 1 Department of Materials Science and Engineering, Norwegian University of Science and Technology (NTNU), 7491 Trondheim, Norway; thomas.brt@hotmail.it (T.B.); ragnhild.aune@ntnu.no (R.E.A.); 2 Hydro Aluminum, Romsdalsvegen 1, 6600 Sunndalsøra, Norway; shahid.akhtar@hydro.com

In the original publication [1], there was a mistake in Table 6 as published. Table 6 had colors correlating the different CFFs to each other, but the table in the published version is empty. The corrected Table 6 appears below.

The scientific conclusions will be unaffected. This correction was approved by the Academic Editor. The original publication has also been updated.

## Figures and Tables

**Table 6:** The corresponding CFF of 30, 50, and 60 PPI from supplier B and supplier C with the Grades 30, 50, 65, and 80 from supplier A.

		Supplier B			Supplier C		
		PPI 30	PPI 50	PPI 60	PPI 30	PPI 50	PPI 60
Supplier A	Grade 30						
	Grade 50						
	Grade 65						
	Grade 80						
